# Supplementary material for: Pyruvate Dehydrogenase Kinase 1 inhibition mediated oxidative phosphorylation enhancement in cartilage promotes osteoarthritis progression
Source: BMC Musculoskelet Disord. 2023 Jul 20;24:597. doi: 10.1186/s12891-023-06585-6 (PMC10357736; doi:10.1186/s12891-023-06585-6)
Supplement: Supplementary file 1 — Additional file 1. [file 12891_2023_6585_MOESM1_ESM.zip › MF.Gsea.1653627316242/GOMF_SINGLE_STRANDED_DNA_BINDING.html]

Details for gene set GOMF\_SINGLE\_STRANDED\_DNA\_BINDING[GSEA]

|  || Dataset | 55457\_collapsed\_to\_symbols.cls.cls #OA\_versus\_normal.cls.cls #OA\_versus\_normal\_repos |
| Phenotype | cls.cls#OA\_versus\_normal\_repos |
| Upregulated in class | OA |
| GeneSet | GOMF\_SINGLE\_STRANDED\_DNA\_BINDING |
| Enrichment Score (ES) | 0.26028177 |
| Normalized Enrichment Score (NES) | 1.220935 |
| Nominal p-value | 0.13239437 |
| FDR q-value | 0.50202036 |
| FWER p-Value | 1.0 |
Table: GSEA Results Summary

  

Fig 1: Enrichment plot: GOMF\_SINGLE\_STRANDED\_DNA\_BINDING      
 Profile of the Running ES Score & Positions of GeneSet Members on the Rank Ordered List

  

| SYMBOL | TITLE | RANK IN GENE LIST | RANK METRIC SCORE | RUNNING ES | CORE ENRICHMENT || 1 | RPA3 | replication protein A3 [Source:HGNC Symbol;Acc:HGNC:10291] | 13 | 1.159 | 0.0312 | Yes |
| 2 | POLR2G | RNA polymerase II subunit G [Source:HGNC Symbol;Acc:HGNC:9194] | 183 | 0.830 | 0.0410 | Yes |
| 3 | STN1 | STN1 subunit of CST complex [Source:HGNC Symbol;Acc:HGNC:26200] | 193 | 0.827 | 0.0633 | Yes |
| 4 | PCBP1 | poly(rC) binding protein 1 [Source:HGNC Symbol;Acc:HGNC:8647] | 197 | 0.823 | 0.0859 | Yes |
| 5 | RPA2 | replication protein A2 [Source:HGNC Symbol;Acc:HGNC:10290] | 339 | 0.727 | 0.0951 | Yes |
| 6 | MCM5 | minichromosome maintenance complex component 5 [Source:HGNC Symbol;Acc:HGNC:6948] | 351 | 0.724 | 0.1143 | Yes |
| 7 | TSN | translin [Source:HGNC Symbol;Acc:HGNC:12379] | 372 | 0.717 | 0.1327 | Yes |
| 8 | TREX1 | three prime repair exonuclease 1 [Source:HGNC Symbol;Acc:HGNC:12269] | 393 | 0.709 | 0.1508 | Yes |
| 9 | MLH1 | mutL homolog 1 [Source:HGNC Symbol;Acc:HGNC:7127] | 497 | 0.660 | 0.1611 | Yes |
| 10 | RPA1 | replication protein A1 [Source:HGNC Symbol;Acc:HGNC:10289] | 571 | 0.632 | 0.1729 | Yes |
| 11 | MSH2 | mutS homolog 2 [Source:HGNC Symbol;Acc:HGNC:7325] | 739 | 0.584 | 0.1761 | Yes |
| 12 | MCM2 | minichromosome maintenance complex component 2 [Source:HGNC Symbol;Acc:HGNC:6944] | 888 | 0.546 | 0.1797 | Yes |
| 13 | RAD51B | RAD51 paralog B [Source:HGNC Symbol;Acc:HGNC:9822] | 893 | 0.545 | 0.1945 | Yes |
| 14 | SSBP2 | single stranded DNA binding protein 2 [Source:HGNC Symbol;Acc:HGNC:15831] | 964 | 0.526 | 0.2036 | Yes |
| 15 | MCM6 | minichromosome maintenance complex component 6 [Source:HGNC Symbol;Acc:HGNC:6949] | 1131 | 0.490 | 0.2043 | Yes |
| 16 | SMC2 | structural maintenance of chromosomes 2 [Source:HGNC Symbol;Acc:HGNC:14011] | 1190 | 0.479 | 0.2130 | Yes |
| 17 | POT1 | protection of telomeres 1 [Source:HGNC Symbol;Acc:HGNC:17284] | 1340 | 0.451 | 0.2139 | Yes |
| 18 | RAD51AP1 | RAD51 associated protein 1 [Source:HGNC Symbol;Acc:HGNC:16956] | 1381 | 0.446 | 0.2232 | Yes |
| 19 | PARK7 | Parkinsonism associated deglycase [Source:HGNC Symbol;Acc:HGNC:16369] | 1406 | 0.442 | 0.2336 | Yes |
| 20 | MCM10 | minichromosome maintenance 10 replication initiation factor [Source:HGNC Symbol;Acc:HGNC:18043] | 1410 | 0.442 | 0.2456 | Yes |
| 21 | POLR3C | RNA polymerase III subunit C [Source:HGNC Symbol;Acc:HGNC:30076] | 1504 | 0.429 | 0.2503 | Yes |
| 22 | RAD51 | RAD51 recombinase [Source:HGNC Symbol;Acc:HGNC:9817] | 1581 | 0.416 | 0.2559 | Yes |
| 23 | ERCC4 | "ERCC excision repair 4, endonuclease catalytic subunit [Source:HGNC Symbol;Acc:HGNC:3436]" | 1696 | 0.400 | 0.2581 | Yes |
| 24 | JCHAIN | joining chain of multimeric IgA and IgM [Source:HGNC Symbol;Acc:HGNC:5713] | 1805 | 0.384 | 0.2603 | Yes |
| 25 | TERF1 | telomeric repeat binding factor 1 [Source:HGNC Symbol;Acc:HGNC:11728] | 1950 | 0.367 | 0.2592 | No |
| 26 | IGHM | immunoglobulin heavy constant mu [Source:HGNC Symbol;Acc:HGNC:5541] | 2241 | 0.335 | 0.2458 | No |
| 27 | POLA1 | "DNA polymerase alpha 1, catalytic subunit [Source:HGNC Symbol;Acc:HGNC:9173]" | 2300 | 0.327 | 0.2504 | No |
| 28 | MYEF2 | myelin expression factor 2 [Source:HGNC Symbol;Acc:HGNC:17940] | 2465 | 0.307 | 0.2461 | No |
| 29 | NABP2 | nucleic acid binding protein 2 [Source:HGNC Symbol;Acc:HGNC:28412] | 2551 | 0.297 | 0.2477 | No |
| 30 | APTX | aprataxin [Source:HGNC Symbol;Acc:HGNC:15984] | 2702 | 0.279 | 0.2437 | No |
| 31 | CTC1 | CST telomere replication complex component 1 [Source:HGNC Symbol;Acc:HGNC:26169] | 2894 | 0.261 | 0.2361 | No |
| 32 | ERCC1 | "ERCC excision repair 1, endonuclease non-catalytic subunit [Source:HGNC Symbol;Acc:HGNC:3433]" | 2948 | 0.255 | 0.2390 | No |
| 33 | REXO4 | "REX4 homolog, 3'-5' exonuclease [Source:HGNC Symbol;Acc:HGNC:12820]" | 2953 | 0.255 | 0.2458 | No |
| 34 | SSBP1 | single stranded DNA binding protein 1 [Source:HGNC Symbol;Acc:HGNC:11317] | 3038 | 0.246 | 0.2460 | No |
| 35 | SETMAR | SET domain and mariner transposase fusion gene [Source:HGNC Symbol;Acc:HGNC:10762] | 3216 | 0.229 | 0.2386 | No |
| 36 | XPC | "XPC complex subunit, DNA damage recognition and repair factor [Source:HGNC Symbol;Acc:HGNC:12816]" | 3278 | 0.222 | 0.2400 | No |
| 37 | RADX | "RPA1 related single stranded DNA binding protein, X-linked [Source:HGNC Symbol;Acc:HGNC:25486]" | 3293 | 0.221 | 0.2450 | No |
| 38 | SMC4 | structural maintenance of chromosomes 4 [Source:HGNC Symbol;Acc:HGNC:14013] | 3574 | 0.196 | 0.2286 | No |
| 39 | SSBP3 | single stranded DNA binding protein 3 [Source:HGNC Symbol;Acc:HGNC:15674] | 3619 | 0.192 | 0.2305 | No |
| 40 | MCM3 | minichromosome maintenance complex component 3 [Source:HGNC Symbol;Acc:HGNC:6945] | 3675 | 0.188 | 0.2314 | No |
| 41 | RBBP8 | "RB binding protein 8, endonuclease [Source:HGNC Symbol;Acc:HGNC:9891]" | 3739 | 0.182 | 0.2315 | No |
| 42 | BRCA2 | BRCA2 DNA repair associated [Source:HGNC Symbol;Acc:HGNC:1101] | 3853 | 0.170 | 0.2274 | No |
| 43 | MAPT | microtubule associated protein tau [Source:HGNC Symbol;Acc:HGNC:6893] | 3976 | 0.158 | 0.2223 | No |
| 44 | NEIL3 | nei like DNA glycosylase 3 [Source:HGNC Symbol;Acc:HGNC:24573] | 4006 | 0.156 | 0.2243 | No |
| 45 | TDP2 | tyrosyl-DNA phosphodiesterase 2 [Source:HGNC Symbol;Acc:HGNC:17768] | 4124 | 0.144 | 0.2192 | No |
| 46 | DMC1 | DNA meiotic recombinase 1 [Source:HGNC Symbol;Acc:HGNC:2927] | 4149 | 0.142 | 0.2212 | No |
| 47 | ERCC5 | "ERCC excision repair 5, endonuclease [Source:HGNC Symbol;Acc:HGNC:3437]" | 4380 | 0.122 | 0.2067 | No |
| 48 | PURA | purine rich element binding protein A [Source:HGNC Symbol;Acc:HGNC:9701] | 4514 | 0.112 | 0.1994 | No |
| 49 | WDR48 | WD repeat domain 48 [Source:HGNC Symbol;Acc:HGNC:30914] | 4735 | 0.092 | 0.1847 | No |
| 50 | TDP1 | tyrosyl-DNA phosphodiesterase 1 [Source:HGNC Symbol;Acc:HGNC:18884] | 4832 | 0.085 | 0.1796 | No |
| 51 | BLM | BLM RecQ like helicase [Source:HGNC Symbol;Acc:HGNC:1058] | 4843 | 0.084 | 0.1811 | No |
| 52 | TERF2 | telomeric repeat binding factor 2 [Source:HGNC Symbol;Acc:HGNC:11729] | 5095 | 0.065 | 0.1633 | No |
| 53 | HMGB2 | high mobility group box 2 [Source:HGNC Symbol;Acc:HGNC:5000] | 5272 | 0.050 | 0.1510 | No |
| 54 | HMCES | "5-hydroxymethylcytosine binding, ES cell specific [Source:HGNC Symbol;Acc:HGNC:24446]" | 5402 | 0.040 | 0.1420 | No |
| 55 | TP53 | tumor protein p53 [Source:HGNC Symbol;Acc:HGNC:11998] | 5511 | 0.032 | 0.1344 | No |
| 56 | SMARCA1 | "SWI/SNF related, matrix associated, actin dependent regulator of chromatin, subfamily a, member 1 [Source:HGNC Symbol;Acc:HGNC:11097]" | 5723 | 0.014 | 0.1183 | No |
| 57 | SAMHD1 | SAM and HD domain containing deoxynucleoside triphosphate triphosphohydrolase 1 [Source:HGNC Symbol;Acc:HGNC:15925] | 5790 | 0.009 | 0.1134 | No |
| 58 | MCM4 | minichromosome maintenance complex component 4 [Source:HGNC Symbol;Acc:HGNC:6947] | 6003 | -0.005 | 0.0970 | No |
| 59 | FUBP3 | far upstream element binding protein 3 [Source:HGNC Symbol;Acc:HGNC:4005] | 6115 | -0.014 | 0.0887 | No |
| 60 | RAD50 | RAD50 double strand break repair protein [Source:HGNC Symbol;Acc:HGNC:9816] | 6474 | -0.040 | 0.0618 | No |
| 61 | HROB | homologous recombination factor with OB-fold [Source:HGNC Symbol;Acc:HGNC:28460] | 6542 | -0.045 | 0.0578 | No |
| 62 | RAD51D | RAD51 paralog D [Source:HGNC Symbol;Acc:HGNC:9823] | 6815 | -0.064 | 0.0383 | No |
| 63 | FUBP1 | far upstream element binding protein 1 [Source:HGNC Symbol;Acc:HGNC:4004] | 7018 | -0.081 | 0.0248 | No |
| 64 | RBMS1 | RNA binding motif single stranded interacting protein 1 [Source:HGNC Symbol;Acc:HGNC:9907] | 7121 | -0.090 | 0.0193 | No |
| 65 | FAM111A | FAM111 trypsin like peptidase A [Source:HGNC Symbol;Acc:HGNC:24725] | 7243 | -0.099 | 0.0126 | No |
| 66 | WBP11 | WW domain binding protein 11 [Source:HGNC Symbol;Acc:HGNC:16461] | 7312 | -0.103 | 0.0102 | No |
| 67 | LONP1 | "lon peptidase 1, mitochondrial [Source:HGNC Symbol;Acc:HGNC:9479]" | 7531 | -0.121 | -0.0035 | No |
| 68 | RTF1 | "RTF1 homolog, Paf1/RNA polymerase II complex component [Source:HGNC Symbol;Acc:HGNC:28996]" | 7597 | -0.127 | -0.0051 | No |
| 69 | SMARCAL1 | "SWI/SNF related, matrix associated, actin dependent regulator of chromatin, subfamily a like 1 [Source:HGNC Symbol;Acc:HGNC:11102]" | 7686 | -0.133 | -0.0082 | No |
| 70 | SMC6 | structural maintenance of chromosomes 6 [Source:HGNC Symbol;Acc:HGNC:20466] | 8043 | -0.158 | -0.0317 | No |
| 71 | TERF2IP | TERF2 interacting protein [Source:HGNC Symbol;Acc:HGNC:19246] | 8054 | -0.159 | -0.0280 | No |
| 72 | PMS2 | "PMS1 homolog 2, mismatch repair system component [Source:HGNC Symbol;Acc:HGNC:9122]" | 8172 | -0.169 | -0.0325 | No |
| 73 | SUB1 | SUB1 regulator of transcription [Source:HGNC Symbol;Acc:HGNC:19985] | 8200 | -0.171 | -0.0298 | No |
| 74 | LRPPRC | leucine rich pentatricopeptide repeat containing [Source:HGNC Symbol;Acc:HGNC:15714] | 8249 | -0.175 | -0.0287 | No |
| 75 | CRY2 | cryptochrome circadian regulator 2 [Source:HGNC Symbol;Acc:HGNC:2385] | 8536 | -0.196 | -0.0456 | No |
| 76 | HNRNPA2B1 | heterogeneous nuclear ribonucleoprotein A2/B1 [Source:HGNC Symbol;Acc:HGNC:5033] | 8576 | -0.200 | -0.0431 | No |
| 77 | RNF138 | ring finger protein 138 [Source:HGNC Symbol;Acc:HGNC:17765] | 8639 | -0.204 | -0.0423 | No |
| 78 | RAD23B | "RAD23 homolog B, nucleotide excision repair protein [Source:HGNC Symbol;Acc:HGNC:9813]" | 8777 | -0.215 | -0.0471 | No |
| 79 | MCM9 | minichromosome maintenance 9 homologous recombination repair factor [Source:HGNC Symbol;Acc:HGNC:21484] | 8781 | -0.215 | -0.0413 | No |
| 80 | RAD23A | "RAD23 homolog A, nucleotide excision repair protein [Source:HGNC Symbol;Acc:HGNC:9812]" | 9028 | -0.234 | -0.0540 | No |
| 81 | POU4F1 | POU class 4 homeobox 1 [Source:HGNC Symbol;Acc:HGNC:9218] | 9300 | -0.256 | -0.0681 | No |
| 82 | YBX1 | Y-box binding protein 1 [Source:HGNC Symbol;Acc:HGNC:8014] | 9623 | -0.285 | -0.0854 | No |
| 83 | MSH3 | mutS homolog 3 [Source:HGNC Symbol;Acc:HGNC:7326] | 9796 | -0.301 | -0.0905 | No |
| 84 | PCBP2 | poly(rC) binding protein 2 [Source:HGNC Symbol;Acc:HGNC:8648] | 9815 | -0.302 | -0.0835 | No |
| 85 | HNRNPU | heterogeneous nuclear ribonucleoprotein U [Source:HGNC Symbol;Acc:HGNC:5048] | 9847 | -0.306 | -0.0774 | No |
| 86 | CDC45 | cell division cycle 45 [Source:HGNC Symbol;Acc:HGNC:1739] | 9982 | -0.319 | -0.0790 | No |
| 87 | MCM7 | minichromosome maintenance complex component 7 [Source:HGNC Symbol;Acc:HGNC:6950] | 10032 | -0.325 | -0.0738 | No |
| 88 | NABP1 | nucleic acid binding protein 1 [Source:HGNC Symbol;Acc:HGNC:26232] | 10052 | -0.326 | -0.0663 | No |
| 89 | RECQL | RecQ like helicase [Source:HGNC Symbol;Acc:HGNC:9948] | 10124 | -0.333 | -0.0625 | No |
| 90 | IGHMBP2 | immunoglobulin mu DNA binding protein 2 [Source:HGNC Symbol;Acc:HGNC:5542] | 10248 | -0.346 | -0.0626 | No |
| 91 | DHX9 | DExH-box helicase 9 [Source:HGNC Symbol;Acc:HGNC:2750] | 10627 | -0.386 | -0.0814 | No |
| 92 | CNBP | CCHC-type zinc finger nucleic acid binding protein [Source:HGNC Symbol;Acc:HGNC:13164] | 10707 | -0.394 | -0.0766 | No |
| 93 | HSF1 | heat shock transcription factor 1 [Source:HGNC Symbol;Acc:HGNC:5224] | 11189 | -0.456 | -0.1015 | No |
| 94 | HSPD1 | heat shock protein family D (Hsp60) member 1 [Source:HGNC Symbol;Acc:HGNC:5261] | 11427 | -0.492 | -0.1064 | No |
| 95 | TOP1 | DNA topoisomerase I [Source:HGNC Symbol;Acc:HGNC:11986] | 11828 | -0.555 | -0.1223 | No |
| 96 | RECQL4 | RecQ like helicase 4 [Source:HGNC Symbol;Acc:HGNC:9949] | 11927 | -0.578 | -0.1139 | No |
| 97 | NUCKS1 | nuclear casein kinase and cyclin dependent kinase substrate 1 [Source:HGNC Symbol;Acc:HGNC:29923] | 11945 | -0.581 | -0.0991 | No |
| 98 | TWNK | twinkle mtDNA helicase [Source:HGNC Symbol;Acc:HGNC:1160] | 12069 | -0.612 | -0.0917 | No |
| 99 | RAD52 | "RAD52 homolog, DNA repair protein [Source:HGNC Symbol;Acc:HGNC:9824]" | 12134 | -0.627 | -0.0793 | No |
| 100 | POLR2H | "RNA polymerase II, I and III subunit H [Source:HGNC Symbol;Acc:HGNC:9195]" | 12167 | -0.635 | -0.0641 | No |
| 101 | HMGB1 | high mobility group box 1 [Source:HGNC Symbol;Acc:HGNC:4983] | 12170 | -0.636 | -0.0466 | No |
| 102 | RAD54L | RAD54 like [Source:HGNC Symbol;Acc:HGNC:9826] | 12204 | -0.646 | -0.0312 | No |
| 103 | HNRNPA1 | heterogeneous nuclear ribonucleoprotein A1 [Source:HGNC Symbol;Acc:HGNC:5031] | 12356 | -0.693 | -0.0238 | No |
| 104 | RPA4 | replication protein A4 [Source:HGNC Symbol;Acc:HGNC:30305] | 12406 | -0.709 | -0.0079 | No |
| 105 | DDX11 | DEAD/H-box helicase 11 [Source:HGNC Symbol;Acc:HGNC:2736] | 12607 | -0.803 | -0.0013 | No |
| 106 | TOP3A | DNA topoisomerase III alpha [Source:HGNC Symbol;Acc:HGNC:11992] | 12707 | -0.867 | 0.0151 | No |
Table: GSEA details [plain text format]

  

Fig 2: GOMF\_SINGLE\_STRANDED\_DNA\_BINDING      
 Blue-Pink O' Gram in the Space of the Analyzed GeneSet

  

Fig 3: GOMF\_SINGLE\_STRANDED\_DNA\_BINDING: Random ES distribution      
 Gene set null distribution of ES for **GOMF\_SINGLE\_STRANDED\_DNA\_BINDING**
